# Stroke care in West Bohemia – personalised medicine approach

**DOI:** 10.1186/1878-5085-5-S1-A83

**Published:** 2014-02-11

**Authors:** Jiri Polivka, Jiri Polivka Jr, Vladimir Rohan, Petr Sevcik, Ondrej Topolcan

**Affiliations:** 1 Department of Neurology, Faculty of Medicine in Pilsen, Charles University in Prague and University Hospital Pilsen, Czech Republic; 2 Department of Histology and Embryology, Faculty of Medicine in Pilsen, Charles University in Prague, Czech Republic; 3 Biomedical Centre, Faculty of Medicine in Pilsen, Charles University in Prague, Czech Republic; 4 Department of Nuclear Medicine, Immunoanalytic Laboratory, University Hospital Pilsen, Czech Republic

## 

Cerebrovascular disease is the third main cause of death and the leading cause of disablement in the Czech Republic. The incidence of stroke is higher than in Western European countries and is one of causes of lower average age in this country. The data of 15880 stroke patients who entered into the IKTA stroke register from 13 Czech stroke centres in the year 2010 and 2011 indicates higher proportion of stroke recurrence and unfavourable patient risk profile than in other Western European countries. Three major risk factors well–defined are arterial hypertension (identified in 86.2% of patients), dyslipidemia (58.2%) and diabetes mellitus (34.9% of stroke patients). Three or more risk factors were found in 80.7% of stroke patients. The incidence of vascular risk factors in stroke patients is significantly higher than stated in the literature and this unfavourable risk profile may be the main cause of the high incidence of stroke and its recurrence in Czech Republic. Stroke is a typical heterogeneous entity. In April 2010 the Ministry of Health published a document “Cerebrovascular Care in the Czech Republic, Constitution of Stroke Centres”. 11 Complex Cerebrovascular Centres and 23 Cerebrovascular Centres covering the whole area of the Czech Republic were constituted with intension to admit, diagnose and, if necessary, treat all new stroke patients, who had been formerly treated in the departments of internal medicine of any hospitals in the country. The aim is to provide complex, optimal and personalised care in acute phase of stroke, provide early rehabilitation and provide or propose optimal second prevention. Faculty Hospital Pilsen incorporates a long-term experience with stroke care and is one of two hospitals where patients with ischaemic stroke were first treated with intravenous thrombolysis in our country. The stroke care is coordinated by the Department of Neurology with its Stroke Unit and Stroke Team. The cooperation with neuroradiology, neurosurgery, cardiology, biochemical and immunoanalytic laboratories, pathology, genetics and some other specialities in Faculty Hospital and in Faculty of Medicine in Pilsen of Charles University in Prague is well established as well as the cooperation with Emergency Medical Service. Due to these facts West Bohemia region with 589 thousands of habitants has only one Complex Cerebrovascular Centre and no one or two smaller Cerebrovascular Centres as is usual in other regions. The advantage of this situation is the concentration of all stroke patients into the best equipped facility. Due to this fact stroke patients in West Bohemia are well diagnosed and treated. Exact data (numbers of thrombolytic, neurosurgical therapy, neuroradiological interventions and their proportion to all stroke patients) are presented. Special research project “Study of Variety Stroke Biomarkers in Acute Stroke Patients in the Context of Personalised Medicine“, supported by Czech Ministry of Health is being solved in our centre from the year 2011. Its main goal is to test the effectiveness of variety of stroke biomarkers and implement them into the personalised stroke care.

Supported by Ministry of Health, Czech Republic - conceptual development of research organization (Faculty Hospital Pilsen - FNPl, 00669806) and by the project ED2.1.00/03.0076 from European Regional Development Fund.

